# Simulation-based procedure training (SBPT) in rarely performed procedures: a blueprint for theory-informed design considerations

**DOI:** 10.1186/s41077-022-00205-4

**Published:** 2022-05-08

**Authors:** David Gent, Ranjev Kainth

**Affiliations:** 1 Faculty of Life Sciences and Medicine, King’s College London, London, UK; 2 St George’s University Hospital NHS Foundation Trust, London, UK; 3 Simulation and Interactive Learning (SaIL) Centre, Guy’s and St Thomas’ NHS Foundation Trust, London, UK

**Keywords:** Simulation-based procedure training, Fidelity, Part-task, Simulation design, Educational theory; Cardiology, Pericardiocentesis, Mastery learning, Evaluation

## Abstract

Simulation-based procedure training is now integrated within health professions education with literature demonstrating increased performance and translational patient-level outcomes. The focus of published work has been centered around description of such procedural training and the creation of realistic part-task models. There has been little attention with regards to design consideration, specifically around how simulation and educational theory should directly inform programme creation. Using a case-based approach in cardiology as an example, we present a blueprint for theory-informed simulation-based procedure training linking learning needs analysis and defining suitable objectives to matched fidelity. We press the importance of understanding how to implement and utilise task competence benchmarking in practice, and the role of feedback and debriefing in cycles of repeated practice. We conclude with evaluation and argue why this should be considered part of the initial design process rather than an after-thought following education delivery.

**Supplementary Information:**

The online version contains supplementary material available at 10.1186/s41077-022-00205-4.

## Introduction

Simulation-based education as a modality is used in various guises: team-based training; human factors specific education; familiarisation of new environments, protocols and procedures; and most recently, to combat rare events such as the coronavirus pandemic [1–3]. Evidence for effectiveness, measured by improvements to patient safety and cost-effectiveness in health systems continues to emerge [4]. One area which has an expanding research base is the use of simulation in the training of specific procedures [5, 6].

### Evidence to practice gap: implementation challenges for educators

Simulation-based procedure training (SBPT) is now firmly integrated into health professions curriculums [6–8] with evidence suggesting it can be used either alongside or in replacement of traditional clinical experience for both low-stakes routine procedures [9, 10] and high-stakes emergency procedures [11–14]. There is heterogeneity in reported outcomes of SBPT with some studies reporting improvement in high-level translational outcomes [15–18] such as reduced intensive care costs and infection rates, and others focusing on lower level outcomes such as time taken to perform a procedure [19, 20]. In such research, publication is centred on data analysis and often lacks sufficient detail regarding educational design, theoretical considerations and implementation. Consequently, this becomes problematic for the healthcare community as they are unable to learn from the work undertaken to replicate and adapt design principles for other procedural-based simulations [21] in their own context, particularly those which are rare and infrequently described in the simulation literature.

A gap thus exists for educators around how high-stakes and rare procedural training should be optimally designed and conducted. The design of SBPT must be reflective of local contextual factors such as learners, faculty, cost and resources which can impact design, immediate measurable outcomes, skill decay and patient safety. In this paper, we provide a blueprint for SBPT which is intended to function as a guide for educators wishing to design and implement procedural training. Using pericardiocentesis as a ‘high-stakes rare-procedure’ case example, we press the importance of focusing on the underlying theoretical rationale for design decisions to optimise participant learning.

### Current approaches to procedural training and curriculum design

**Fig. 1:**
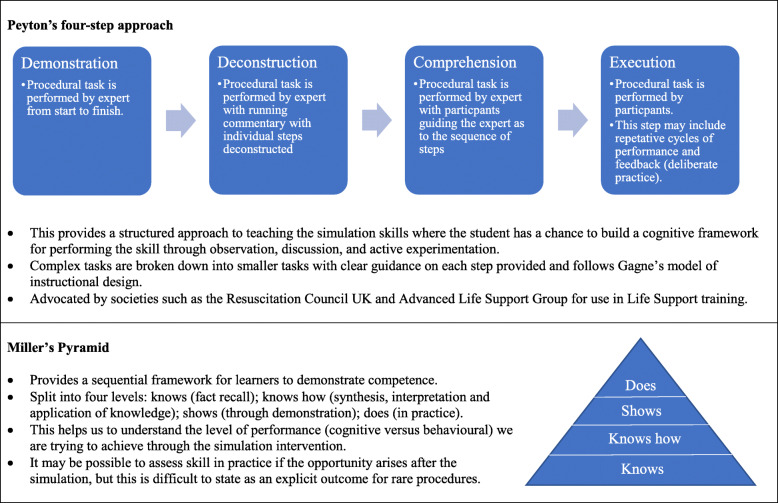
Contemporary approaches to procedural training. We have highlighted two traditional theoretical approaches to delivering procedural training. Both of these models focus on deconstruction of the task either via small steps and subordinate tasks (Peyton’s four-step approach) [23] or by distinguishing cognitive knowledge and behavioural knowledge (Miller’s Pyramid) [24]

Traditional approaches to procedural training focus on a 'see-one, do-one, teach-one' methodology where there is an assumption that competence immediately follows observation [22], failing to recognise the risk to patient safety as few people are competent to independently perform a procedure after one observation. More contemporary structured approaches (Fig. 1) includes Peyton’s four-step approach which aims for educators to deconstruct the activity and scaffold learning, and frameworks such as Miller’s pyramid which guide the educator in thinking about the level of performance we want learners to attain [23, 24]. Theory-informed design in SBPT goes beyond these frameworks and is multi-faceted encompassing discrete, sequential items for educators to consider when trying to maximise learning yield.

Several authors have published curriculum development tools with explicitly defined steps with Kern et al. [25] and Sawyer et al. [26] both providing comprehensive approaches (Table 1). Other sources focus more on the pure delivery of teaching psychomotor skills highlighting how sessions can be stratified based on the pre-procedural skill of the learner and desired learning outcomes [27].

**Table 1:** Established curriculum development frameworks. The proposed SBPT Blueprint incorporates parts of both frameworks alongside additional design considerations centred around education theory

Kern et al.’s [25] six steps	(i) Identification of a problem and a general needs assessment; (ii) targeted needs assessment; (iii) goals and objectives; (iv) educational strategies; (v) implementation; (vi) evaluation and feedback.	• This approach acknowledges that curriculum development is dynamic with multiple interacting components and interplay between steps. For example, availability of resources will have an impact on the learning objectives. • It streamlines curriculum development attempting to align targeted goals and objectives with implementation and evaluation. • The aim is to improve the efficiency and effectiveness of teaching. • The Kern approach is widely applicable to different fields of teaching.
Sawyer et al.’s [26] six steps	(i) Pre-simulation didactic learning (ii) observation of the procedure; (iii) deliberate practice; (iv) proof of competency prior to performing the skill on a patient; (v) doing the procedure on patients; (vi) maintenance through continued practice.	• Simulation training is split explicitly into cognitive and psychomotor phases with an expectation of adequate theoretical knowledge before simulator practice. • This approach promotes skill maintenance through continued practice but does not explore repetition intervals. • It is more directive than the Kern approach and focused more on the design of procedural skill training. • It explicitly includes a human performance element which decreases its utility for teaching rarely performed procedures.

**Fig. 2:**
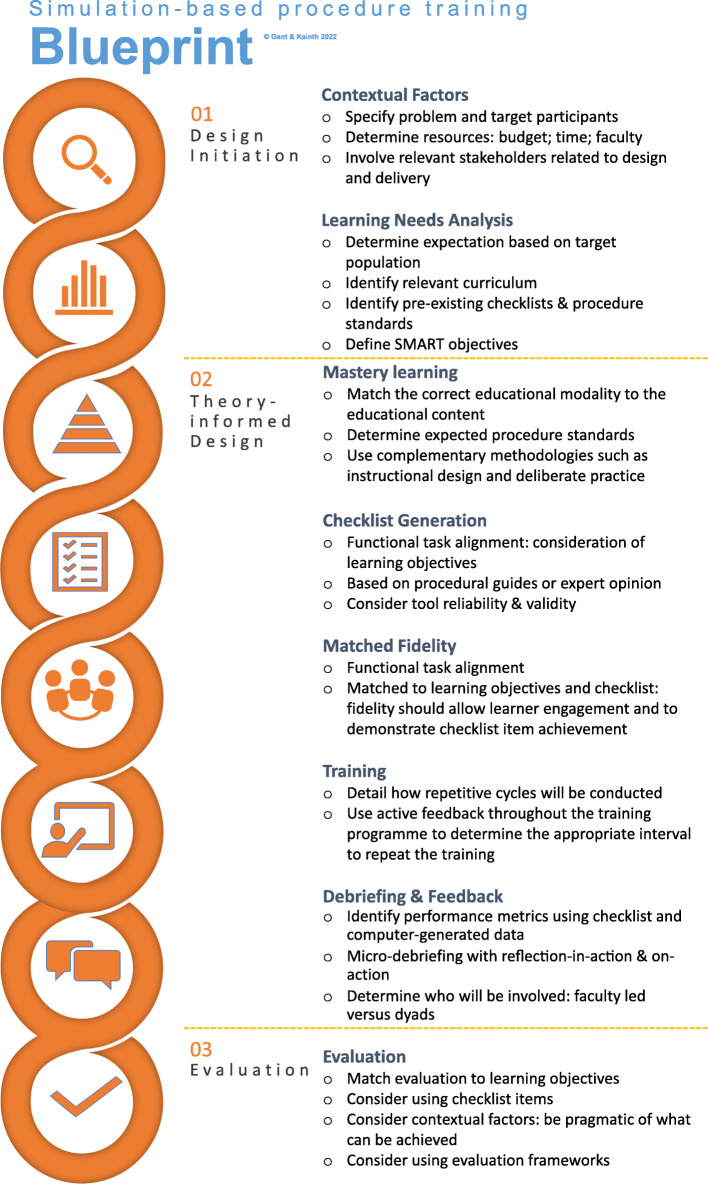
SBPT Blueprint. This is an example of how the SBPT blueprint functions in practice. Each element follows from the previous element and considers relevant contextual factors. The content of each element directly influences overall design and should be expanded. For example, under mastery learning, a pre-requisite would be for all participants to be familiar with the equipment and where applicable, time for this must be integrated into the programme or provided in an alternative fashion (e.g. online education) if required. Factors such as resources and evaluation which are often ill-considered can be discussed at the onset by mapping out a comprehensive blueprint

Our approach to curriculum design for SBPT is particularly suited to rarely performed procedures, uses concepts from a variety of these approaches and includes additional elements such as fidelity considerations, skills decay and debriefing and feedback considerations. This curriculum design blueprint, presented in Fig. 2, outlines the sequential theory-informed design elements which educators should consider when designing SBPT in their own context.

## SBPT Blueprint

### Stage 1: Design initiation

This is perhaps the most critical stage in the design process where contextual factors are identified and specified such as target participants, available resources, and key stakeholders to be involved. It is important that technical and logistical issues are addressed in parallel to SBPT content design as often, successful implementation is dependent upon both. We refer the reader to the guidelines by Khan et al. for an outline of these elements in more detail [28].

#### Learning needs analysis

A learning needs analysis (LNA) should be carried out beforehand to determine which areas of the curriculum would benefit from simulation training. The LNA is not limited to individual skills but can encompass communication, teamworking and other non-technical skills [29–34]. Many different tools can be used for the LNA with the exact tool chosen to suit the scope of the analysis and the context of the teaching (Table 2). Care needs to be taken to include all relevant stakeholders and enough time needs to be allocated for the assessment to be completed [35, 36]. It is important to note that a LNA can be generic, such as identifying pericardiocentesis amongst many different procedures, or more specific by identifying elements of a procedure which require particular attention such as calculating blood flow velocity or obtaining particular imaging views when undertaking echocardiography; different approaches to LNA can provide different information.

**Table 2:** Mechanisms to undertake a learning needs analysis

• Interviews, group discussions or focus groups • Surveys: participant and patient • Participant reflections, for example from portfolio entries and logbook • Self-assessment or peer assessment against established standards • Peer review and observation (including using simulation) • Themes from audit or research reports • Relevant publications—for example, from professional bodies, government and departmental reports • Critical incident review and safety reports • Review of relevant curriculum and Delphi assessment to identify specific needs of a procedure	

##### Case study: undertaking a learning needs assessment using participant survey.

A recent electronic survey of European cardiology trainees found high levels of reported self-confidence performing temporary pacing wire and central venous cannula insertion compared to pericardiocentesis or transoesophageal echocardiography [37]. This result was driven by rarity of certain procedures and variable exposure to acute cardiac pathology coupled with scarce simulation-based training [37] mirroring other specialities. Interestingly pericardiocentesis was ranked lowest by trainees in terms of comfort yet, it is also the procedure in which trainees report having the least simulation training [37]. SBPT is being used within cardiology although these endeavours lack information around theory-informed design [38], negatively impacting empirical research in cardiology-specific SBPT [6]. The curriculum gap and lack of theory-informed design make pericardiocentesis a good choice for a worked case example in SBPT (Box 1).

### Stage 2: Theory-informed design

#### Mastery learning as an overarching pedagogical framework

The initial steps within the SBPT Blueprint design process are to determine whether the SBPT is constructively aligned to curriculum objectives, defining learning outcomes based upon the LNA, and deciding which overarching pedagogical framework will allow these to be delivered [39]. Appropriate curriculum integration enhances learning when compared to ad-hoc simulation which is facilitated by ensuring that the learning objectives chosen are Specific, Measurable, Achievable, Realistic and Time-related [40, 41]. For a rarely performed lifesaving intervention like pericardiocentesis, each specialty registrar is required to reach a universally high level of competence to perform the task; achievable through a mastery learning (ML) approach [5, 42–44].

It is important to note that we are not exclusively suggesting a ML approach for all procedures; an appropriate pedagogical framework should be linked to the underlying objectives. There is, however, emerging evidence that clinical outcomes between SBPT using traditional instruction compared with demonstration and unsupervised independent practice are not significantly different suggesting a ML approach would be best suited for all procedure-based training to keep learning in line with explicit standards [45]. Instructional design and deliberate practice (DP) are core components of ML that aid the practical implementation of the framework [46, 47] centred around upskilling individuals to a desired standard with task-specific feedback, facilitated by use of a checklist. Table 3 outlines these theories and explains how they inform SBPT design for our case study. These theories do not operate as ‘stand-alone’ and instead should be seen as synergistic explanatory frameworks in understanding how participants learn, enabling educators to design interventions to optimise desired outcomes.

#### Generating a checklist

When we implement the instructional design model, a reference teaching and assessment checklist is required which outlines the core technical elements. To save time at this stage it is important to identify whether suitable validated teaching checklists already exist [12]. Even if a pre-existing checklist is not validated it could form a basis for a checklist thus preventing duplication.

If no checklists exist a functional task alignment analysis [48] can identify the technical steps required to complete a skill and give an insight into what tasks and processes the simulator will be required to perform. Checklist items can be further informed from curriculum documents/published procedural guides [48] and expert panel consensus statements [48].

Ideally, checklist items need to be independently validated and passing standards agreed in line with the intended learning outcomes. The Delphi methodology focuses on iterative rounds of checklist review by experts aiming to mould the teaching tool and is context dependent [30–32]. It may require input from a range of stakeholders (expert and non-expert) and is time-consuming with fatigue occurring in later rounds [29, 36]. A judgement needs to be made by the designers regarding which portions of the checklist are essential for task completion and traditional standard-setting approaches such as the Angoff method may be useful here [49]. The process of checklist generation and functional task alignment, dependent upon local context and learners, is critical as this determines what degree of realism is required to achieve the checklist items. We have provided an example checklist for pericardiocentesis as Supplementary Table 1.

#### Fidelity and learning

Traditionally, fidelity had been viewed as a unidimensional concept with increasingly complex technology being synonymous with an increased representation of reality. Subsequent theories have split fidelity into four main components—environmental, mechanical (or engineering), psychological and sociological fidelity [50–52], although other domains exist. As an example, a difficult communication scenario would require high sociological fidelity (e.g. with actors or standardised patients) compared to suturing where banana skin may suffice for novice learners and animal tissue for advanced learners representing different degrees of tactile feedback (mechanical fidelity) adjusted according to the learner. The key, then, is selecting the most appropriate design features to match the intended learning objectives which themselves are tailored to learners and the context.

Choosing the level of fidelity is important as it can impact on both the cost of delivering the teaching and learner engagement. A systematic review looking at clinical performance as an outcome demonstrated higher fidelity simulations are associated with a small but non-significant gain in performance outcomes compared to lower fidelity simulation which is offset by increased cost [52]. The reason for the lack of difference is thought to be multifactorial: context may not be as important as assumed; high psychological fidelity may be generated even with low mechanical fidelity design; and, lower fidelity simulation may lead to reduced cognitive load (Table 3). Paradoxically there may even be detrimental effects of high-fidelity simulation when teaching novices as it may promote unsafe over-confidence [53, 54]. Despite this, higher fidelity simulation may be more appropriate for advanced learners to give an adequate level of psychological fidelity. Whilst there are many commercially available simulators, high- and low-fidelity simulators, particularly for procedural skills, can be created from readily accessible material, such as gelatine-based and 3D printing, at low cost [11, 13, 19, 55, 56].

Determining the simulation design, which may consider multiple different fidelity domains, is further impacted by cognitive load. In our case study, participants are novices at performing pericardiocentesis, and according to the cognitive load theory, they would benefit from simulation design with a high intrinsic and germane load and a reduced extrinsic load so they are not overwhelmed during the task (Table 3) [57]. Approaches such as virtual reality, augmented reality and mixed reality may provide realistic training experiences, especially when these are combined with tactile feedback in procedural skills. However, they are associated with a significant increase in cognitive load and may be best reserved for advanced trainees who require more complex simulator feedback for engagement [58, 59]. These design choices and trade-off between fidelity and cognitive load are rooted in the initial needs analysis and functional task alignment [62] and attempting to reconstruct the entirety of clinical reality for all learners is inappropriate. Rather, we strive for constructive alignment between learning needs, learning objectives and fidelity matched to benchmarked standards in the form of a checklist.

**Table 3:** The key educational frameworks which influence simulation-based procedural training and their relationship to our simulation design

Educational theory	Relationship to pericardiocentesis simulation design
**Mastery learning** [5, 43, 44]	
• A framework for acquisition of skills across multiple domains incorporating behaviourism and cognitivism. • Consists of (i) baseline learner assessment (ii) defining learning objectives in units of varying difficulty (iii) defining mastery standards (iv) educational activity (v) formative assessment and feedback against pre-set standard (vi) repetitive practice until standard met (vii) movement to next educational unit. • Highly protocolised—all learners aim to reach uniform competence in set units before moving on to the next unit. • Incorporates deliberate practice with pre-defined passing standard. • Impacted by time-limitation and may not be appropriate for all interventions (e.g. those without easily measurable outcomes).	• Pericardiocentesis requires a universally high level of competence for all trainees. • It is highly protocolised and lends itself well to a mastery-based approach. • A baseline level for learners is assumed based upon requirements to enter training programme. • Learning units consist of internal anatomy; surface anatomy; equipment familiarisation and setup; procedure completion; post-procedure management.
**Instructional design** [46, 60]	
• Focuses on deconstructing a complex task or skill and rebuilding it from smaller components. • Expectation of achieving competence in each of these subordinate tasks (educational units). • The knowledge may be theoretical knowledge such as anatomy and landmarks and psycho-motor knowledge. • Fragmenting the information allows the teaching to be delivered in chunks with repetitive cycles, debriefing and feedback.	• Pericardiocentesis can be a complex task for learners and benefits from breaking the procedure down into small tasks. • These subordinate tasks build the checklist. • Information can be given in stages and re-tested. For example, pre-course learning material given to establish theoretical knowledge which is then tested at the beginning of the session.
**Deliberate practice** [43, 47]	
• Originated from research on training in music performance. • Deliberate practice occurs in cycles: defined unit goal–practice–feedback. • Involves motivated learners, informative feedback, performance monitoring and error correction. • Can be seen as the ‘educational activity’ in mastery learning programmes. • Feedback is critical to correct errors in performance until the passing standard is met. • Potentially time consuming due to the variability in time taken to reach the passing standard.	• Pericardiocentesis requires all learners to reach a minimum competency standard. • Deliberate practice would facilitate this and provides support to learners who take longer to master the skills. • Debriefing and feedback is facilitated by the checklist and may be undertaken by dyad learners or course faculty depending on the educational unit.
**Cognitive-load theory** [57, 61]	
• Helps us to understand how people learn as there is a limit on how much new information people can consume at one time. • Cognitive load factors include: o *intrinsic load*—difficulty level of the task; o *germane load*—inherent difficulties aiding learning; o *extrinsic load*—external factors impeding learning. • High intrinsic and germane load and low extrinsic load promote consolidation of long-term memories from working memory. • These factors influence design by shaping the required fidelity of the simulation. • See Reedy [57] and Fraser et al. [61] for a deeper understanding of the theory and the application to simulation design.	• For pericardiocentesis we want a focus on upskilling novices to perform a manual task by providing a cognitive framework. • We need to limit the extraneous load. For example, excluding actors playing allied healthcare professionals. • The complexity of the situation (simulation scenario design) can be increased to increase extraneous load to engage more advanced learners. • Design should be based on achieving the intended learning objectives whilst providing enough stimulus for learning.

#### Skills decay: contextualising programme delivery

An area of contention when designing simulation courses is when to repeat the intervention. There is no consensus on the durability of acquired skills or the retention interval, and various studies looking at skills decay have conflicting results. There are reports of skills being retained for 14 months in some studies with others finding evidence of decreased performance as little as 6 months after simulation training [12, 63–65]. When simulation re-test has been stratified into domains (affective, psychomotor or cognitive) no decrease in any specific domain has been found [66]. These studies are complicated by small sample sizes, heterogenous designs and various confounding factors. A recent scoping review by Donoghue et al. agreed that these were consistent issues making aggregation of results difficult [67]. The authors did conclude, however, that studies which were informed by theory, specifically, DP and ML, improved educational outcomes with less skill decay compared to other education delivery methods. This accounts for the advocacy of inclusion of DP and ML in the 2020 American Heart Association guidelines for resuscitation training [68].

Potential methods to augment skill retention include distributing the teaching session over more sessions than originally planned [69] and giving students access to simulators with dedicated unsupervised training time after the first supervised training session [70]. The consistent messages from various studies are perhaps unsurprising: there is a signal towards increased proficiency and skill retention with increasing seniority of the learners and students who have repeated training sessions show increased proficiency in task performance [9, 20, 71].

In summary, skills decay is an evolving area within SBPT that requires a greater evidence base to provide firm guidance for curriculum design. It is a complex topic that is dependent on multiple factors including those related to the task (complexity of the task, frequency that the procedure will be performed in practice), learner (novice vs. advanced) and healthcare setting (available resources and curriculum integration within the healthcare system). Until more research emerges on the optimal retention interval this design consideration should be determined at the curriculum development and learning needs analysis stages.

#### Appropriate feedback and debriefing

Feedback and debriefing are crucial steps for skill acquisition. They traditionally occur at the end of a simulation exercise but can occur during the simulation. Feedback is information given about the comparison between the observed performance and the desired outcome and debriefing is an interactive discussion that facilitates a reflection on the performance [72, 73].

There are various feedback and debriefing strategies available. In the context of ML, there is evidence that micro-debriefing improves performance by facilitating DP and attainment of the minimum passing standard [63, 74]. There had been initial concern that continued feedback during the performance can lead to cognitive overload [75] but repeated feedback has been shown to improve the efficiency of SBPT as assessed by procedural outcomes [76]. Micro-debriefs can be employed either whilst the simulation is running (in-action, e.g. what organs have you identified to avoid puncturing with the needle) providing direct feedback on tasks being performed or following a brief pause in the simulation scenario (on-action, e.g. the angle of needle entry was too deep) providing feedback on tasks just performed [73].

Micro-debriefing using reflection on-action is a core component of Rapid Cycle Deliberate Practice (RCDP) [74, 77] and alongside reflection in-action this can be a useful strategy to teach novice learners who lack a frame of reference. RCDP is a relatively novel approach that involves immediate feedback on actions in a coaching style, increasing the amount of time spent on DP. For more advanced trainees an approach using reflective pauses may be appropriate as it allows an exploration of the learners’ frame of reference thus increasing their engagement and allowing them to bring their own experience into the teaching session [78]. This facilitates discussion rather than unidirectional feedback and promotes double-loop learning (providing the underlying rationale for an action) which is more effective than single-loop learning (simply correcting an action) [79, 80].

Regardless of which feedback and debriefing strategy is chosen we need to create a learning environment where learners take risks and are open to feedback and engage in debriefing [81]. Psychological safety and mutual respect can be generated through highlighting the importance of specific feedback, what type of feedback strategy you will use during the SBPT and making it clear that perfection is not expected from the start [82]. There is evidence of benefit in peer-based dyadic teaching in SBPT; this is a cost-effective strategy that is associated with a significant increase in the efficiency of SBPT [83, 84]. If this is the chosen strategy, learners should be made aware of the value of peer evaluation at the beginning of the SBPT [10]. These strategies are in addition to already established frameworks to foster psychological safety in simulation, for example, as outlined by Rudolph et al. [81] and Kolbe et al. [85].

Overall, it is important to think about the type of feedback, source and timing when designing the SBPT in order to plan when and how the feedback and debriefing will occur [60]. Traditional end-of-task feedback and debriefing still has its uses such as generating post-task discussion in scenarios with a specific ethical dilemma or scenarios where there are multiple potential outcomes.

#### Online learning

There is a growing interest in the use of online videos to aid clinical skills teaching supported by high-quality evidence [86]. Online videos can play a role in multiple parts of SBPT such as allowing students to review a recording of the procedure being performed prior to attending training, creating more time for DP; providing a source for feedback and discussion; and, allowing the student to review the steps of a procedure in the future once the SBPT session is complete. Despite the advantages of online learning there are potential issues such as variation in the trainee’s ability to access the content, patient confidentiality considerations if real patients are involved, and variation in the quality of online learning videos. Use of video will thus be dependent upon local resources and recognising how and why recorded material is to be used; it should not be assumed, as with other design components, that simply inserting an additional pedagogical modality results in increased learning.

### Stage 3: Evaluation considerations

When evaluating individual SBPT, it can be difficult to demonstrate that ML in simulation can lead to high-level (T3, T4) translational outcomes if the focus of the intervention is a rarely performed clinical procedure. An alternative may be to demonstrate a reduction in healthcare costs which would be a strong argument in a taxpayer-funded healthcare system such as the National Health Service (NHS), although understandably this is seldom the main goal of a ML programme.

The need for simulation courses for rare procedures is driven by the infrequency with which these procedures are encountered in clinical practice. Unfortunately, this limits the amount of data you can collect to demonstrate higher level translational outcomes unless you have an extensive follow-up time. Because of these restrictions, the goal of many ML programmes for rare procedures will be to produce improvements in competence within the simulation setting on re-testing (T1). This does not represent a failure in evaluation by providing weak evidence, but it is a pragmatic approach to the evaluation of a rare procedure, and we encourage similar rational approaches to evaluation. In comparison, for procedures frequently undertaken in clinical practice such as lumbar puncture, intubations and catheter insertions, evaluation may be conducted to look at improvements in specific domains, including patient-level outcomes and hospital-wide benefits such as reduced costs.

Whilst data collected at evaluation will be constructively aligned to learning outcomes and can be considered robust, evaluation of SBPT should not be limited to positivist metrics and can include qualitative approaches which may provide insights regarding impact on clinical practice and patient safety. The key is to match available resources with intended aims of evaluation, and here, toolkits such as the King’s College London Evaluation Practice Toolkit [87] provide useful direction.

Despite evidence showing that ML leads to improved patient care and clinical outcomes, the quality of reporting from studies implementing mastery-based simulation programmes is not uniform [5, 88]. In order to address this, Cohen et al. outlined a 38 item Reporting Mastery Education Research in Medicine (ReMERM) guideline to provide educators, authors and journal editors with a gold-standard framework for reporting mastery interventions [89]. When authors report their findings using this framework it allows a detailed comparison and aggregation of studies in a systematic review and provides a framework for other authors to replicate design aspects of the simulation intervention as it calls on authors to provide a detailed description of the simulation intervention in the methodology section.

## Conclusions: sharing our journeys

We have provided a comprehensive blueprint for simulation designers engaging in SBPT focusing on theoretical issues whilst attending to contextual and practical influences (Fig. 2). Our core steps for curriculum design relevant to SBPT encompass three main phases. The design initiation phase consists of defining the problem, understanding local contextual factors including determining available resources and stakeholder identification. This, coupled with a detailed LNA will determine the course objectives, specific to the learners. Logistical elements need to be considered from the beginning and will often span the entire design process and be influenced by decisions around checklist formation, fidelity and training interval.

In the second phase, an appropriate pedagogical framework should form the scaffold for design. The learners and learning objectives will define the expected standards and development of a checklist in a mastery learning approach. The key next step is to determine how the required knowledge can be achieved; specifically, what simulation setup, or fidelity, is required to simultaneously meet the standards, engage the learners and avoid cognitive overload. The next step, which is dependent upon factors such as the number of faculty and group size, is how cycles of practice can be delivered with corresponding debriefing and feedback. Consideration then needs to be given to training intervals to determine how frequently over the course of a year or training programme learners will be involved in the training again.

Alongside designing the intervention, realistic goals for evaluation need to be set. This again will be contextual and based upon resources, time and what type of outcome measures are feasible. Approaches such as focus groups, interviews and surveys may be a cost-effective option, especially if the aim of evaluation is to refine the course in the early stages before attempting to measure patient-level outcomes.

It is through rigorous adherence to design principles where we, as simulation educators, provide justice to our learners and ultimately to patients. Often, the simulation community report success with varying translational outcomes or descriptive pieces outlining novel simulation interventions. We call for detailed, theory-informed SBPT design to be made available so others can replicate, adapt, contextualise and share in the success.

## Supplementary Information

**Additional file 1.**

## Data Availability

The following additional material is available: Supplementary file one: Pericardiocentesis example checklist.

## Abbreviations

SBPT: Simulation-based procedure training
LNA: Learning needs analysis
ML: Mastery learning
DP: Deliberate practice
