# Supplementary material for: Simulation-based procedure training (SBPT) in rarely performed procedures: a blueprint for theory-informed design considerations
Source: Adv Simul (Lond). 2022 May 8;7:13. doi: 10.1186/s41077-022-00205-4 (PMC9079208; doi:10.1186/s41077-022-00205-4)
Supplement: Supplementary file 1 — Additional file 1. [file 41077_2022_205_MOESM1_ESM.docx]

| **Checklist Item** | **Achieved**  **(Y/N)** | **No. of attempts to achieve** | **Subunits – these need to be completed in full to progress to the next component** |
| --- | --- | --- | --- |
| Correctly identify patient and ask to complete the WHO checklist |  |  | Subunit 1 – *Whitespace for written feedback notes* |
| Wash hands |  |  |  |
| Ultrasound used to confirm presence of effusion and entry point |  |  |  |
| Wash hands |  |  |  |
| Get trolley and clean |  |  |  |
| Set up aseptic field on the trolley and open pericardiocentesis Kit (the Boston and Cook kits are most common) |  |  | Subunit 2 |
| Will need to ask assistant for saline ampoules and lignocaine ampoules (1% or 2%) |  |  |  |
| Wash hands and apply gown and sterile gloves |  |  |  |
| Clean the skin and apply drapes |  |  |  |
| Apply drapes |  |  |  |
| Repeat ultrasound is required at this point and they need to ask for the sterile probe cover |  |  | Subunit 3 |
| Flush the introducer needle, dilator and catheter with saline. Put the guidewire introducer in place |  |  |  |
| Inject 10mls of 2% lignocaine first via a green needle into the subcutaneous space and the tract |  |  |  |
| Attach 10ml syringe with agitated saline onto the introducer needle) |  |  |  |
| Introduce needle in a slow and controlled manner supporting the needle at the entry point until pericardial fluid is aspirated |  |  |  |
| Injected agitated saline and rescreen with echocardiography to ensure the needle is in the pericardial space |  |  |  |
| Remove the syringe and insert the guide wire (up to 10-15cm) |  |  |  |
| Apply incision to the skin and dilate the tract with two dilators of increasing size with a rotational movement to ensure good tract expansion |  |  | Subunit 4 |
| Apply catheter over guide wire and measure the pressure in the pericardium |  |  |  |
| Secure the drain to the drainage bag |  |  |  |
| Apply dressing. Learners are not expected to suture the drain in place during the simulation |  |  |  |
| Confirm you would measure the pericardial pressure once the effusion has been drained |  |  | Subunit 5 |
| Complete an example notes entry describing the procedure and the post-procedure instructions to the nursing staff including the need for continuous cardiac monitoring, chest x-ray and trans-throracic echocardiogram. |  |  |  |
